# Adsorption behavior of Eu(III) on partially Fe(III)- or Ti(IV)-coated silica

**DOI:** 10.1186/1556-276X-7-51

**Published:** 2012-01-05

**Authors:** Hee-Jung Im, Kyoung Kyun Park, Euo Chang Jung

**Affiliations:** 1 Nuclear Chemistry Research Division, Korea Atomic Energy Research Institute, 150 Deokjin-dong, Yuseong-gu, Daejeon, 305-353, Republic of Korea

**Keywords:** partially coated silica, Ti(IV) coating effects, enhanced adsorption, surface complexation.

## Abstract

The adsorption behavior of Eu(III) onto silica surface, which was partially coated with Fe(III) or Ti(IV), was investigated to determine Fe(III) or Ti(IV) effects on the surface reaction of lanthanides on mineral surfaces in groundwater. Compared with a parallel uncoated silica, the Fe(III)-coated silica did not enhance the adsorption of Eu(III). However, enhanced adsorption of Eu(III) on the Ti(IV)-coated silica was observed by increasing the amount of Ti(IV) on the silica surface.

## Introduction

There has been great interest in the immobilizations and adsorption mechanisms of various toxic ions in aqueous solutions by using silica-based sorbents [1-4]. The adsorption reaction of a metal ion onto a metal (hydr)oxide surface is explained in terms of surface complexation. Besides free metal ions, hydrolyzed or complexed species [5], or even the colloidal species can be adsorbed [6]. Surface precipitation may occur even in a concentration below the surface site saturation [7]. Frequently, experimental evidence indicates surface nucleation of metal hydroxides [8].

Due to their ubiquity in soils and sediments and high specific surface area, iron or titanium hydroxides (Fe or Ti oxides) around silicate minerals may play a role in the migration of actinides in groundwater. The interactions of actinides on immobile solid surfaces are important processes that determine retardation during transport. Usually, Eu(III) is considered to be an adequate chemical analogue of radiotoxic nuclides, Am(III) and Cm(III). These actinides consist mainly of long-lived nuclides that emit alpha radiation, and their radioactivity continues for several hundred thousands of years [9].

The following studies have been reported. Fe-modified silica gel was investigated as an adsorbent for humic acids employing an electrostatic binding to Fe and/or in coordination with Fe by direct substitution of OH and Cl on Fe sites [10]. TiO_2_-coated SiO_2 _synthesized by hydrolysis and condensation of various silicate and titanate precursors has been actively studied as photocatalysts due to its photocatalytic and photovoltaic effects [11]. Eu(III) sorption onto clay minerals was quantitatively modeled with pH ranging from 3 to 10 using cation exchange reactions for Eu(III)/Na(I) and Eu(III)/Ca(II) [12].

However, Eu(III) sorption onto Fe(III)- or Ti(IV)-coated silica has not received as much attention as UO_2_^2+ ^sorption. The aim of this paper is to study the sorption of Eu(III) from an aqueous solution on Fe(III)- or Ti(IV)-coated silica to understand the trace radionuclide migration which occurs in groundwater.

## Experimental section

The chemicals used in this study including silica (Sigma-Aldrich Corporation, St. Louis, MO, USA; particle size 40 to 63 μm; surface area 550 m^2^/g), ferric nitrate [Fe(NO_3_)_3_·9H_2_O], titanium butoxide [Ti(OBu)_4_], 1, 10-phenanthroline [C_12_H_8_N_2_], ethanol [C_2_H_5_OH], toluene [C_6_H_5_CH_3_], and europium(III) oxide [Eu_2_O_3_] were all of high purity and used as received. Perchloric acid [HClO_4_], hydroxylamine hydrochloride [NH_2_OH·HCl], sodium perchlorate [NaClO_4_], sodium hydroxide [NaOH], sulfuric acid [H_2_SO_4_], and hydrofluoric acid [HF] were of analytical grade and used without further purification. NaOH solution was titrated with 0.1 M hydrochloric acid [HCl] standard solution (Merck & Co., Inc., Whitehouse Station, NJ, USA) in the presence of a phenolphthalein indicator.

The dry silica was dispersed in 3.7 M HNO_3 _for one day and washed with distilled water until the wet silica surface was neutral. Finally, the resulting silica was dried in an oven at 120°C for 6 h and stored in a capped bottle after cooling.

The 11.3 mM Eu_2_O_3 _in 20.62 mM HClO_4 _was prepared as a stock solution for the Eu(III) adsorption tests. The metal-ion concentration of the stock solution was determined with inductively coupled plasma - atomic emission spectrometry [ICP-AES] before diluting for the adsorption experiments. All the solutions were handled under a nitrogen gas flow.

Coated, adsorbed, and desorbed metal concentrations were determined with an ultraviolet and visible [UV-vis] absorption spectrophotometer (Cary 3, Varian, Inc., Santa Clara, CA, USA) and an ICP-AES (ULTIMA 2C, HORIBA Jobin Yvon, HORIBA, Ltd., Minami-ku, Kyoto, Japan). A spectrofluorometer (FS-900CD, Edinburgh Instruments, Livingston, U.K.) was used to obtain appropriate fluorescence spectra.

### Partial Fe(III) coating on silica surface

Fe(NO_3_)_3_·9H_2_O (0, 35, 70, 140, 280, and 420 mg) was added with stirring to each silica (50 g) in 500 mL of distilled water. The pH was adjusted to 4.5 with 0.1 M HClO_4 _or 0.1 M NaOH, and each mixture was stirred for 2 h. The partially Fe(III)-coated silica was glass filtered, washed with a pH 4.5 HClO_4 _solution and distilled water three times each, and dried at 120°C for 6 h sequentially.

### Partial Ti(IV) coating on silica surface

Ti(OBu)_4 _was slowly added with stirring to each silica (15 g) in C_2_H_5_OH and C_6_H_5_CH_3_ (1:1) mixed solution until 0, 5, 10, 20, 100, and 200 mM of Ti(IV) was added in the 50 mL total solution. Then, each mixture was stirred for 2 h. The partially Ti(IV)-coated silica was glass filtered, washed with a C_2_H_5_OH and C_6_H_5_CH_3 _(1:1) mixed solution three times, and dried in sequence at 120°C for 6 h.

### Eu(III) adsorption onto Fe(III)- or Ti(IV)-coated silica

In each test, 500 mg of dissimilar Fe(III)- or Ti(IV)-coated silica was placed in a 60-mL beaker, and 20 mL of distilled water was added in the beaker. The total volume of each mixture was adjusted to 50 mL, and the final concentration was 0.1 mM Eu_2_O_3 _in 0.18 mM HClO_4 _with a controlling ionic strength with 0.1 M NaClO_4_. At this point, for the observation of a pH-dependent adsorption, 0.1 M NaOH under the N_2 _gas flow in order to eliminate the remaining CO_2 _in the solution, was properly added to each mixture, and the pH went up to 8 for Eu(III) adsorption tests. The mixture in each polyethylene beaker was stirred for more than 30 min until the pH equilibrium was achieved. The mixture was then analyzed by ICP-AES after being filtered through a 0.1-μm pore-sized membrane filter. Fluorescence of Eu ions on Fe(III)- or Ti(IV)-coated silica was obtained from the sediments.

## Results and discussion

It has been known that ≡Si-OH in silica (SiO_2_) is dissociated into surface ≡Si-O^- ^and free H^+ ^at pH > 3, and as the result, the surface is negatively charged, which is appropriate to incorporate electron-deficient metals to the silica surfaces. Here, Fe(III) or Ti(IV) was primarily fixed on the silica surface through the ≡Si-O-Fe or ≡Si-O-Ti structure. In contrast, Eu(III) ion is easily hydrolyzed [13] and forms insoluble trihydroxide precipitates [14] and polynuclear hydroxo complexes [15]. The hydrolyzed Eu-OH is assumed to be sorbed into Fe(III)- or Ti(IV)-coated silica in aqueous Eu(III) solutions.

Each and every coated Fe ion on the silica surface was stripped using 5 M HCl, and the amount was measured using a UV-vis spectrophotometer by reducing all Fe(III) to Fe(II) with NH_2_OH·HCl for the production of colored Fe(II)-orthophenanthroline complexes (ferroin, (Phen)_3_Fe^2+^), which are sensitive to UV-vis absorption at 510 nm [16]. From the UV-vis absorption spectra, 0, 2.05, 3.81, 7.38, 13.8, and 21.3 μmol/g (Fe/silica) Fe-coated silica was obtained when 0, 35, 70, 140, 280, and 420 mg of Fe(NO_3_)_3_·9H_2_O were added in 50 g of each silica. During this process, the following product is expected:

$$\equiv \text{Si - OH }+\text{ F}{\text{e}}^{\text{3}+}+\;{\text{H}}_{\text{2}}\text{O }\to \equiv \text{Si - O - Fe - OH}$$

**Figure 1:**
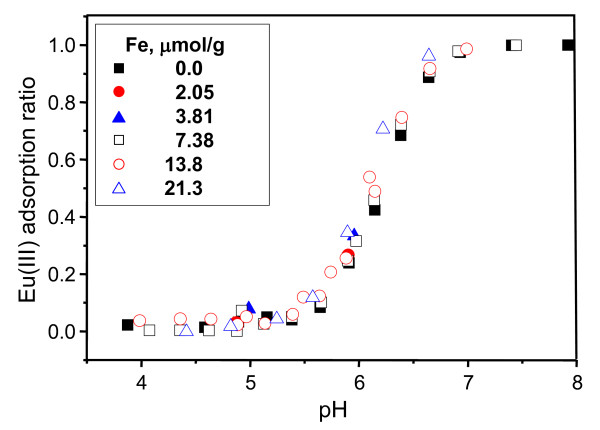
**Eu(III) adsorption on partially Fe(III)-coated silica at a concentration of 0.1 mM Eu(III)**. Eu(III) adsorption ratio is defined as the Eu(III) concentration adsorbed on partially Fe(III)-coated silica versus the initial Eu(III) concentration.

The adsorption of Eu(III) onto the silica at various pH shows no influence of a surface coating by Fe(III) (Figure 1). In other words, Fe(III) coating on silica surfaces did not enhance adsorption of Eu(III) compared with the uncoated silica. The paper written by Pokrovski et al. [17] states, 'at pH > 2.5 in the presence of aqueous silica (*m*_fe _approximately at 0.01 mol/kg), Fe-Fe dimers and trimers shared one or two edges of FeO_6_-octahedra, and silicon tetrahedra linked to two neighboring Fe octahedral via corners'. Due to linkages to the free corners of FeO_6_-octahedra, the number of available sorption sites for Eu(III) in Fe^III^-OH had decreased, and thus, it did not show a significant Fe(III)-coating effect compared with the uncoated silica.

**Figure 2:**
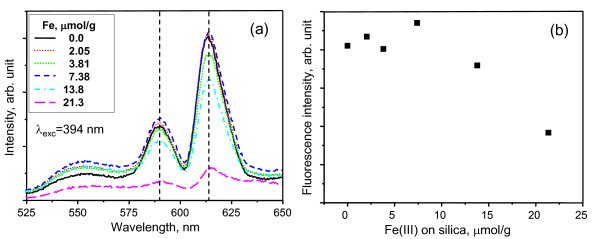
**Fluorescence spectra and intensities of Eu(III) on partially Fe(III)-coated silica**. (**a**) Fluorescence spectra (*λ*_exc _= 394 nm), and (**b**) fluorescence intensities (*λ*_exc _= 394 nm) of Eu(III) on partially Fe(III)-coated silica at 613 nm, according to the amount of coated Fe(III) at pH 7.

**Figure 3:**
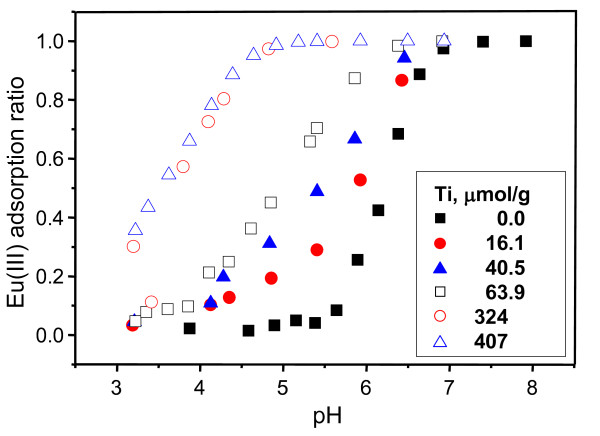
**Eu(III) adsorption on partially Ti(IV)-coated silica at a concentration of 0.1 mM Eu(III)**. Eu(III) adsorption ratio is defined as the Eu(III) concentration adsorbed on partially Ti(IV)-coated silica versus the initial Eu(III) concentration.

However, as shown in Figure 2, the fluorescence decreased with an increased amount of coated Fe(III) even though the shape of the fluorescence spectra did not change. In Figure 2a, the Eu(III) fluorescence spectra excited at 394 nm were scanned from 525 to 650 nm. This range covers the wavelengths corresponding to ^5^D_0 _→ ^7^F_J _(*J *= 0, 1, and 2) transitions, and the peaks at 588 nm and 613 nm correspond to ^5^D_0 _→ ^7^F_1 _and ^5^D_0 _→ ^7^F_2_, respectively. Since the ^5^D_0 _→ ^7^F_1 _transition is allowed in magnetic dipole and its strength is not sensitive to coordination environment, the decrease in the peak intensity can be explained in terms of a quenching effect. The fluorescence intensities of Eu(III) at 613 nm were quantitatively expressed in Figure 2b according to the amount of coated Fe(III). The ^5^D_0 _→ ^7^F_2 _transition should also be affected by the quenching effect as with the ^5^D_0 _→ ^7^F_1 _transition. In contrast, Ti(IV)-coated silica showed higher Eu(III) adsorption capacities while increasing the amount of Ti(IV) on silica surface as shown in Figure 3.

The amount of every coated Ti ion on the silica surface was measured using ICP-AES after a pretreatment process with H_2_SO_4_-HF solution to remove SiO_2_. From the ICP-AES analysis, 0, 16.1, 40.5, 63.9, 324, and 407 μmol/g (Ti/silica) Ti-coated silica was obtained when 0, 5, 10, 20, 100, and 200 mM (in other words, 0, 20, 40, 80, 350, and 700 μmol/g (Ti/silica)) of Ti(OBu)_4 _were added in 15 g of each silica. In this process, the following product is expected:

$$\equiv \text{Si - OH }+\text{ T}{\text{i}}^{\text{4}+}+\;{\text{H}}_{\text{2}}\text{O }\to \equiv \text{Si - O - Ti - OH}$$

The Ti(IV)-coated silica exhibited a stronger binding toward Eu(III) than the uncoated silica, and the preferential binding is considered due to a higher metal Lewis acidity of Ti than Si. The hard Lewis acid, Eu(III), forms more stable complexes with hydroxyl ligands on relatively harder TiO_2 _than those on SiO_2_. The Eu(III) adsorption processes onto the partially Ti(IV)-coated silica involve the combination of Eu(III) hydrolysis and the adsorption of the hydrolysis product, Eu^III^-OH, to produce ≡Si-O-Ti-OH-Eu^III ^and/or ≡Si-O-Ti-O-Eu^III ^in addition to ≡Si-OH-Eu^III ^and/or ≡Si-O-Eu^III^, depending on the pH of the prepared solutions [12].

**Figure 4:**
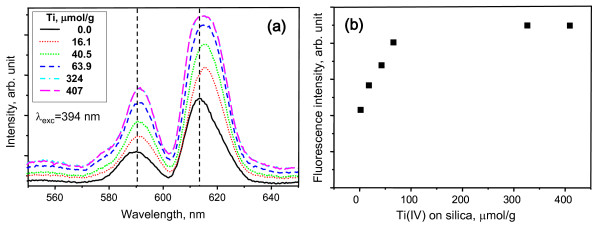
**Fluorescence spectra and intensities of Eu(III) on partially Ti(IV)-coated silica**. (**a**) Fluorescence spectra (*λ*_exc _= 394 nm), and (**b**) fluorescence intensities (*λ*_exc _= 394 nm) of Eu(III) on partially Ti(IV)-coated silica at 613 nm, according to the amount of coated Ti(IV) at pH 7.

The enhanced adsorption of Eu(III) onto the silica coated by Ti(IV) is partially confirmed by observing the increase in fluorescence intensities as increasing the amount of Ti(IV) on the silica surface as shown in Figure 4[18]. The fluorescence intensities of Eu(III) at 613 nm were quantitatively expressed in Figure 4b according to the amount of coated Ti(IV). In the case of Eu(III), the maximum fluorescence peaks corresponding to ^5^D_0 _→ ^7^F_2 _transition at 613 nm were red-shifted in a wavelength nearly 618 nm with an increased coated Ti(IV) (Figure 4a). It suggests the formation of other species with a reduced hydration number such as [≡Si-O-Ti-O]_2_-Eu^III ^or ≡Si-O-Ti-O-Eu^III^-OH. No fluorescence-quenching effect of the increased amount of coated Ti(IV) indicates a similar chemical environment between the species reacting with the titanol and silanol functional groups.

## Conclusions

This study shows an example of foreign ion effects on the adsorption of actinide onto a mineral surface. In the case of the Ti(IV) ion for Eu(III) adsorption onto a silica surface, Ti(IV) enhances the adsorptivity as far as it exists as a surface hydroxide. The enhancement in adsorptivity decreases when the surface hydroxide converts to oxide prior to Eu(III) adsorption. In contrast, Fe(III) coating on silica surfaces did not enhance adsorption of Eu(III), nor were there any changes in fluorescence properties compared with uncoated silica.

## Competing interests

The authors declare that they have no competing interests.

## Authors' contributions

H-JI drafted the manuscript, prepared samples, and acquired various adsorption and spectrographic data. KKP conducted the preparation of samples and analysis of data. ECJ coordinated the interpretation of data. All authors read and approved the final manuscript.
